# Line-scanning confocal microendoscope for nuclear morphometry imaging

**DOI:** 10.1117/1.JBO.22.11.116005

**Published:** 2017-11-11

**Authors:** Yubo Tang, Jennifer Carns, Rebecca R. Richards-Kortum

**Affiliations:** Rice University, Department of Bioengineering, Houston, Texas, United States

**Keywords:** microendoscope, line-scanning, optical sectioning, low-cost, digital light projector, CMOS rolling shutter

## Abstract

Fiber-optic endomicroscopy is a minimally invasive method to image cellular morphology *in vivo*. Using a coherent fiber bundle as an image relay, it allows additional imaging optics to be placed at the distal end of the fiber outside the body. In this research, we use this approach to demonstrate a compact, low-cost line-scanning confocal fluorescence microendoscope that can be constructed for <$5000. Confocal imaging is enabled without the need for mechanical scanning by synchronizing a digital light projector with the rolling shutter of a CMOS camera. Its axial performance is characterized in comparison with a nonscanned high-resolution microendoscope. We validate the optical sectioning capability of the microendoscope by imaging a two-dimensional phantom and *ex vivo* mouse esophageal and colon tissues. Results show that optical sectioning using this approach improves visualization of nuclear morphometry and suggest that this low-cost line-scanning microendoscope can be used to evaluate various pathological conditions.

## 1 Introduction

Endomicroscopy in combination with molecular probes has provided clinicians with a powerful tool to visualize tissue architecture and cellular morphology to investigate disease progression. In probe-based endomicroscopy, a coherent fiber bundle is used to enable microscopic imaging with subcellular resolution through the working channel of a standard endoscope. Due to its minimal invasiveness, it is widely applicable in the evaluation and management of many clinical conditions, such as early detection of neoplasia in the gastrointestinal tract,^1^^–^^6^ cervix,^7^ pancreas,^8^ and lung.^9^ Existing commercial and research platforms, such as the Cellvizio endomicroscopy system (Mauna Kea Technologies, Paris, France)^10^ and the high-resolution microendoscope (HRME),^11^ offer an opportunity to provide real-time histological information.

The coherent fiber bundle used in probe-based endomicroscopy serves as an optical image relay that allows for external implementation of sophisticated opto-mechanical systems at its proximal end. Optical sectioning, for example, can be introduced to increase the axial resolution via structured illumination or confocal scanning.^12^^,^^13^ The resulting benefits, as demonstrated in a range of laboratory and clinical studies,^14^^–^^19^ are manifold. When used with topical staining, optical sectioning has been shown to reduce out-of-focus light and improve image contrast; in applications that require the use of IV staining such as fluorescein, optical sectioning is critical for the rejection of high background signals.^14^^,^^17^^,^^18^ It is also particularly desirable in imaging highly scattering tissues with crowded nuclei, such as in regions of precancer or cancer.^14^^,^^15^ The enhanced ability to resolve individual nuclei can also potentially facilitate development of automated algorithms to diagnose diseases based on cell morphology such as nuclear size, density, and nuclear-to-cytoplasmic area ratio.^20^^,^^21^ In addition, subsurface tissue imaging can be realized in confocal endomicroscopy by incorporating depth focusing objectives at the distal fiber end.^10^^,^^19^^,^^22^

The implementation of optical sectioning, however, usually requires integration of complex optomechanical components to rapidly scan and descan individual fibers in the coherent bundle, which adds to system cost.^23^ Alternatively, the rolling shutter of a CMOS detector can be used to achieve versatile slit detection without the need for a physical aperture.^24^ On the illumination end, a digital light projector (DLP) can be synchronized as a spatial light modulator to project matching illumination lines and perform confocal imaging.^25^^,^^26^ In this work, we present the first demonstration of a line-scanning confocal microendoscope based on a DLP and a CMOS camera without the need for mechanical scanning. We characterized its axial performance in comparison with a nonscanned HRME (or the standard HRME),^2^ and we validated its optical sectioning capability by imaging a two-dimensional (2-D) phantom and *ex vivo* mouse esophageal and colon specimens. The system offers high-resolution endoscopic imaging with optical sectioning and can be built into a compact enclosure at a low cost (<$5000).

## 2 Methods

### 2.1 Optical Setup

The standard HRME is described in detail elsewhere.^11^ Briefly, the HRME probe is a coherent fiber bundle consisting of 30,000 individual fibers with a core-to-core spacing of ∼4  μm and a circular field of view (FOV) of 720  μm (IGN-08/30, Sumitomo Electric Industries). An LED centered at 455 nm (M455L2, Thorlabs, Newton, New Jersey) was used to provide illumination and a scientific CCD camera (Grasshopper 2, FLIR Integrated Imaging Solutions Inc., Richmond, Canada) was used for fluorescence imaging.

**Fig. 1:**
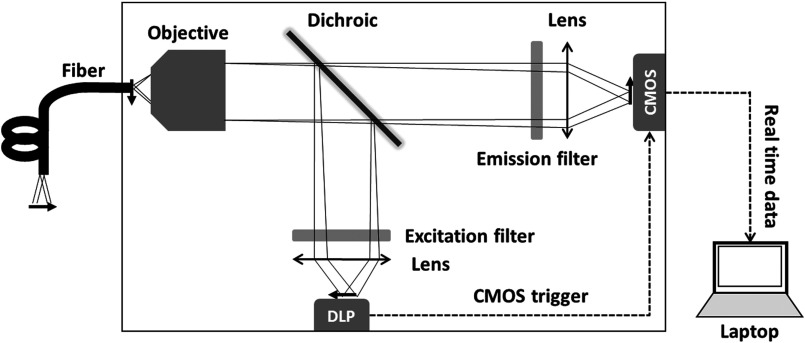
Schematic of the line scanning confocal HRME platform. Solid arrows show directions of scanning on the DLP, CMOS, and fiber surface; dashed arrows show the data flow between the DLP, camera, and laptop. The solid box indicates the optical system enclosure.

In the line-scanning confocal HRME, scanning illumination is provided by a DLP. For confocal detection, a rolling shutter CMOS sensor replaces the CCD camera with a global shutter to offer a versatile electrically controllable detection slit. A schematic of the confocal HRME is shown in Fig. 1. A DLP (LightCrafter 4500, Texas Instrument, Dallas, Texas) was used to program the illumination patterns. The blue LED of the DLP light engine is centered at 448 nm with a 16-nm bandwidth and was used for fluorescence excitation. The projected lines were focused through a collimation condenser (f=125  mm, LA1986, Thorlabs, Newton, New Jersey) and a 10× objective (RMS10X, Thorlabs, Newton, New Jersey) on the proximal fiber end; scanning of the illumination across the fiber was achieved by the sequential projection of illumination line patterns. Fluorescence signal collected by the fiber was imaged with a scientific CMOS sensor (Firefly MV USB 2.0, FLIR Integrated Imaging Solutions Inc., Richmond, Canada) through an imaging lens (f=75  mm, LA1257, Thorlabs, Newton, New Jersey). The optical setup at the proximal end, as shown in the solid box in Fig. 1, was housed in a 400-×355-×150-mm enclosure.

A laptop was used to program the DLP pattern sequence and retrieve the camera images. During image acquisition, a transistor–transistor logic signal was sent from the DLP to trigger the camera exposure; the rolling shutter was synchronized with the DLP sequence to perform confocal imaging.

### 2.2 Synchronization and Confocal Imaging

**Fig. 2:**
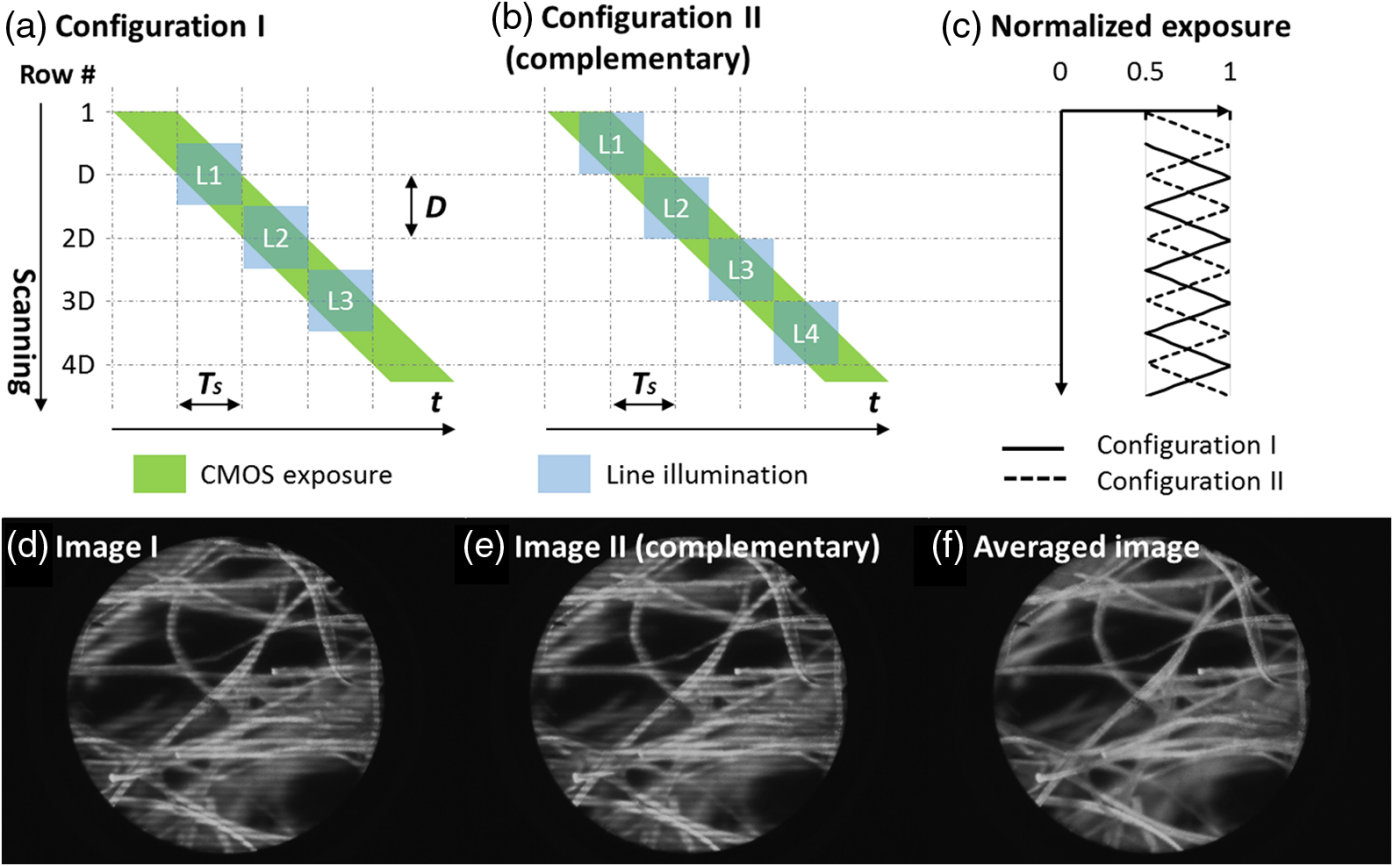
Synchronization of the CMOS and DLP and construction of a confocal image by averaging two complementary images. Imaging configuration I in (a) shows the spatiotemporal coordination of the DLP illumination lines with the CMOS rolling shutter aperture; the discrete nature of DLP scanning combined with the continuous sequential readout of CMOS rows introduces nonuniform exposure among the rows, as revealed in configuration I in (c) and the corresponding image (d). A complementary image (e) can be acquired by shifting the exposure distribution by D/2 in configuration II in (b). A confocal image without illumination artifacts is then constructed by averaging two complementary frames, as shown in (d), (e), and (f). Lens paper was imaged as a target. D, number of rows in the detection aperture; $T_{s}$, CMOS exposure time.

The CMOS sensor offers a rolling shutter that can be used as a versatile slit for confocal detection. The exposure of rows on a CMOS sensor is activated sequentially at a fixed line frequency $f_{L}$(for the Firefly MV USB 2.0, 16231 Hz), as illustrated by the green parallelogram in Fig. 2(a). As exposure begins on each row, photons are acquired for a predefined shutter time $T_{s}$ before the readout takes place. With a fixed line frequency, the number of rows under exposure concurrently (D), and hence the width of the rolling shutter aperture, can be controlled by adjusting the shutter time $T_{s}$:

$$D=T_{s}*f_{L}.$$ (1)

To project line-scanning illumination, the DLP is programmed to perform in pattern sequence mode. Each illumination sequence is saved as a one-bit pattern that can be projected by toggling on and off an array of micromirrors (for the DLPLightCrafter 4500, 912×1140  μm). The LightCrafter 4500 frame buffer supports storage of two 24-bit images, allowing up to 48 continuous illumination sequences during a single frame acquisition. The size and optical magnification of projected illumination lines were adjusted so that they match the aperture width D.

The spatiotemporal coordination between illumination and detection is illustrated in imaging configuration I in Fig. 2(a). As shown in the green parallelogram, the rolling shutter aperture contains D rows undergoing exposure simultaneously and scans across the active FOV (for the Firefly MV USB 2.0,960 rows) row by row. To cover a total number of 960 rows using 48 DLP sequences in a nonoverlapping manner, the illumination lines with a matching size (L1, L2, L3, etc.) are scanned with a step no fewer than 20 rows, each of them centered at rows D, 2−D, 3−D, etc. Meanwhile, the projection of each illumination line is synchronized with the exposure of the pixel row at its center. Illumination line L1, for example, is only projected when its central row (row D) undergoes exposure. The remaining illumination lines are projected in a similar manner without overlap.

The discrete nature of DLP scanning combined with the continuous sequential readout of CMOS rows introduces nonuniform exposure among the rows. The effective exposure time $T_{E}$, defined as the temporal overlap between the CMOS exposure and DLP illumination [the green parallelogram and blue rectangles in Fig. 2(a), respectively], varies for each row along the scanning direction. Specifically, $T_{E}$ is maximal at the illumination central rows [i.e., rows D, 2−D, 3−D, etc. in Fig. 2(a)] and drops by half at the borders of the illumination lines as shown in Fig. 2(c). The resulting image reveals illumination artifacts in Fig. 2(d).

A complementary image can be used to eliminate the illumination artifacts. To capture an image with a complementary $T_{E}$ distribution in Fig. 2(c), the illumination lines are physically shifted by D/2 rows while maintaining the temporal synchronization in imaging configuration II as shown in Fig. 2(b). By averaging the two frames in Figs. 2(d) and 2(e), a confocal image without illumination artifacts is constructed in Fig. 2(f). Both imaging configurations were programmed in the DLP, so the complementary images can be obtained with two consecutive frames. The averaged images were displayed in a MATLAB Graphical User Interface (The MathWorks, Natick, Massachusetts).

### 2.3 Characterization of Axial Sectioning Performance

The optical sectioning capability of the confocal HRME was evaluated and compared with a standard HRME. The axial resolution was also measured as the optical slit size was varied by tuning the shutter time and adjusting the DLP sequences.

To accurately evaluate the optical sectioning performance, a mirror (PF10-03-G01, Thorlabs, Newton, New Jersey) was used as a target. The system was converted into reflection mode to image the mirror by removing the emission filter. The mirror was mounted to a stepper motor (LSA10A-T4, Zaber Technologies, Vancouver, BC, Canada) and was initially placed in gentle contact with the fiber bundle surface. It was then moved away from the fiber in 20-μm increments up to 400  μm. The image intensities were measured at each axial distance. The background signal, mainly from internal reflections, was determined with the mirror removed and subtracted from all measurements. This was repeated for slit widths of 20, 40, 60, 80, and 100 pixel rows, with the 20-pixel slit as the smallest aperture size to scan the entire FOV.

### 2.4 Two-Dimensional Phantom Validation and Ex Vivo Imaging

The optical sectioning performance in fluorescence mode was first evaluated by imaging a 2-D phantom. The fluorescence phantom was developed as previously described.^17^ Briefly, 15  μL of 15-μm fluorescent polystyrene microspheres (F-21010, Thermo Fisher Scientific, Waltham, Massachusetts) in solution were pipetted onto a glass slide, which was then allowed to dry for about 20 min prior to imaging. Similar to the mirror, the 2-D phantom was first imaged in gentle contact with the fiber and then imaged each time the fiber was retreated by 20  μm. This was repeated for slit widths of 20-, 40-, and 60-pixel rows.

The confocal HRME was also evaluated by imaging excised mouse tissue *ex vivo*. Mice were acquired from Jackson Laboratories. Squamous epithelium in the esophagus and columnar epithelium in the colon were imaged after topically staining with proflavine (0.01% w/v in phosphate buffered saline). The contrast in images acquired with the standard and confocal HRME was compared. In squamous epithelium, the nuclear and cytoplasmic regions were manually segmented and the nuclear-to-cytoplasmic signal ratio was calculated. In columnar epithelium, the contrast between the glandular walls and lumens in line scans was quantified and compared. All animal experiments were reviewed and approved by the Institutional Animal Care and Use Committee of Rice University.

## 3 Results

### 3.1 Axial Sectioning Performance

**Fig. 3:**
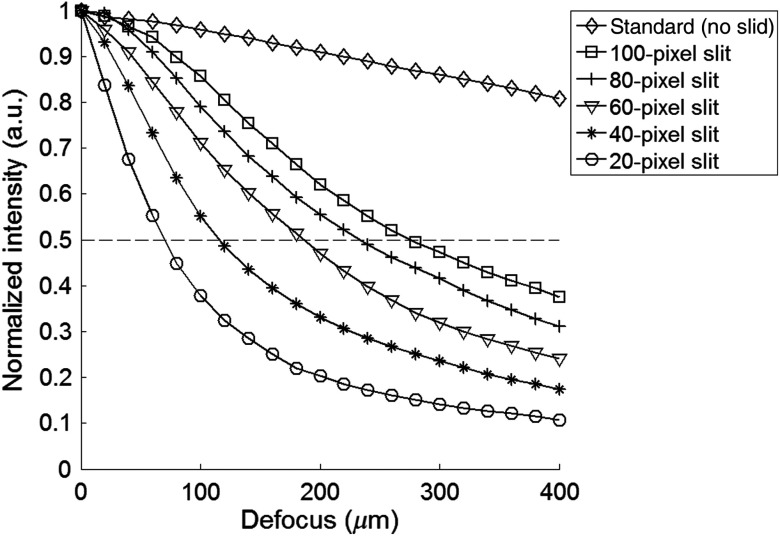
Optical sectioning profiles of the standard HRME (no slit) and confocal HRME (20, 40, 60, 80, and 100-pixel slit). The line scanning confocal configurations significantly improve the axial resolution of the standard HRME. As the slit size increases, the axial performance transits from the confocal to nonconfocal regime. A 20-pixel slit on the CMOS sensor is about 16.5  μm on the fiber surface.

The sectioning profiles with and without the confocal line-scanning are shown in Fig. 3. Without slit detection, the standard HRME demonstrated minimal out-of-focus signal rejection. When the line-scanning was introduced, the background rejection was significantly improved in the confocal HRME. The optical sectioning was most prominent using a 20-pixel slit on the CMOS sensor, which corresponded to about 16.5  μm on the fiber surface; as the confocal aperture size was increased, the axial performance transited from the confocal to the nonconfocal regime.

### 3.2 Two-Dimensional Phantom and Ex Vivo Validation

**Fig. 4:**
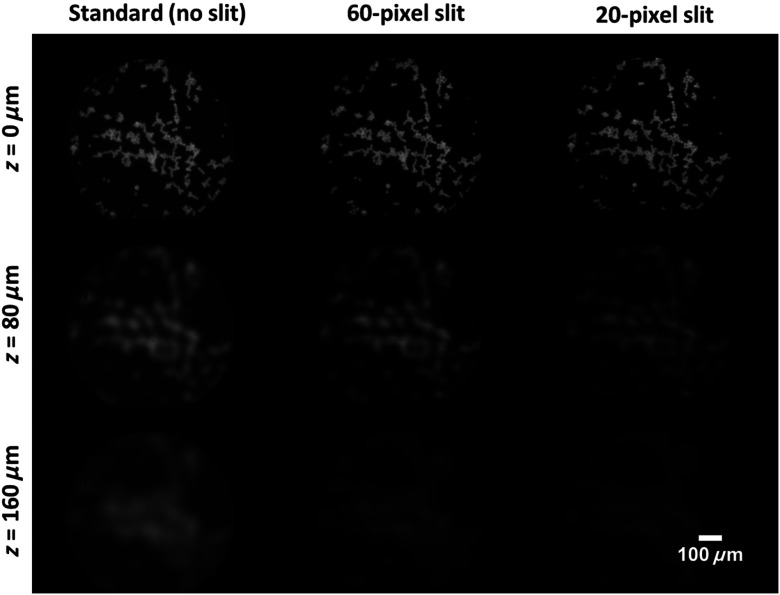
2-D phantom images with varied slit widths. Images acquired at a defocus of 0, 80, and 160  μm are shown for the standard HRME and two confocal configurations (60-pixel and 20-pixel slits). Compared with the image at 0  μm, the corresponding image at 80 and 160  μm revealed signal loss and indicated rejection of background signal. The signal loss was most significant using the 20-pixel slit, suggesting that a smaller detection slit in the confocal configuration improves optical sectioning capability.

The findings above were validated by imaging a monolayer of 15-μm beads in fluorescence mode. In each column of Fig. 4, the image at 0  μm was normalized to itself, and images at greater axial distances were adjusted equally. Compared with the image at 0  μm, the corresponding image at 80 and 160  μm appeared defocused and revealed intensity loss. The signal loss was minimal using the standard HRME (no slit) and was most striking when a 20-pixel slit was used. These findings were consistent with the axial profiles in Fig. 3 and confirmed that a small detection slit improves optical sectioning capability.

**Fig. 5:**
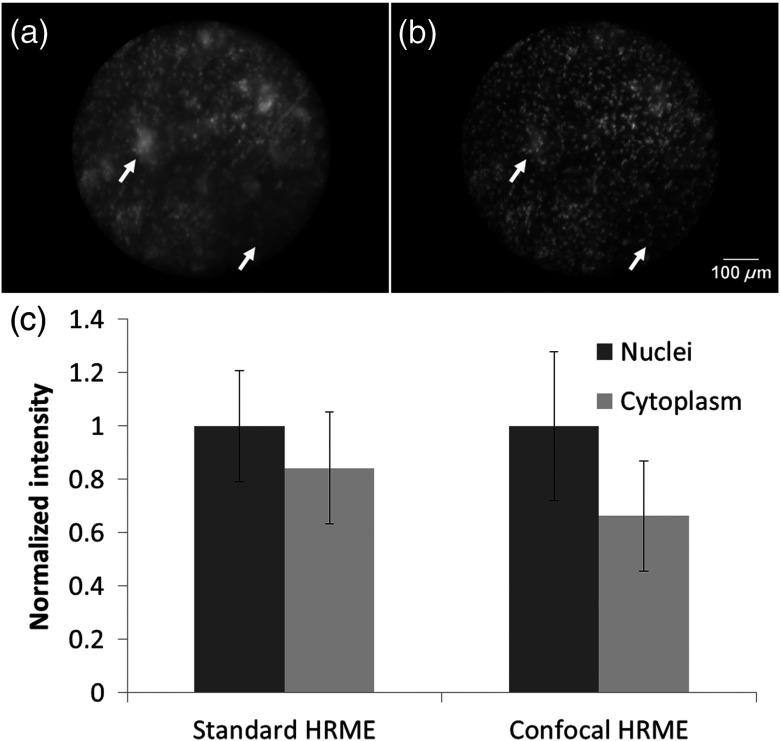
*Ex vivo* images of mouse squamous esophageal epithelium using (a) the standard and (b) confocal HRME. The confocal HRME resolved nuclei with enhanced contrast, especially in regions indicated by the arrows. (c) The normalized intensities of nuclear and cytoplasmic regions are shown; the error bars show the intensity standard deviation in these regions. The resulting ratio of the nuclear-to-cytoplasmic signal in the standard and confocal HRME was 1.19 and 1.51, respectively.

Based on the results above, a 20-pixel slit was used in subsequent evaluation of the imaging performance in highly scattering tissue samples. Figure 5 shows images of mouse squamous esophageal epithelium acquired by the standard (A) and confocal (B) HRME. Both images were normalized for visual comparison. Compared with the standard HRME, the confocal HRME demonstrated improved rejection of out-of-focus signal and enhanced image contrast. The enhancement was most striking in regions indicated by the white arrows, with the confocal image showing clearly defined nuclei that were difficult to resolve in the standard HRME. The difference was quantified by manually segmenting the nuclear and the cytoplasmic regions; the average intensity of nuclear and cytoplasmic regions was normalized and shown in Fig. 5(c). The ratio between the nuclear-to-cytoplasmic signal in the standard and confocal HRME was 1.19 and 1.51, respectively.

**Fig. 6:**
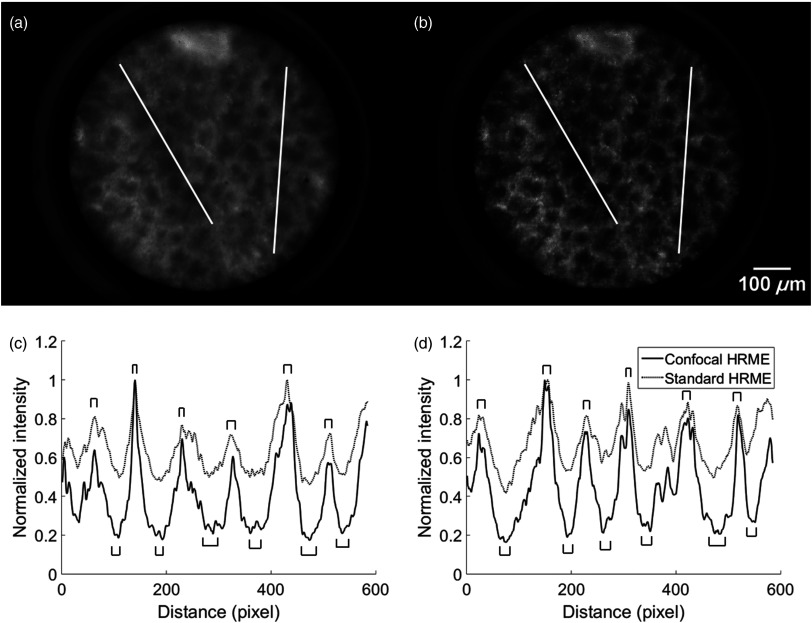
*Ex vivo* images of mouse columnar epithelium using (a) the standard and (b) confocal HRME. Profiles are shown for the line scans on the (c) left and (d) right. The brackets indicate the glandular walls and lumens. The resulting gland-to-lumen ratio was 101%±26% higher in the confocal than the standard HRME (1.57±0.25 in standard and 3.20±0.84 in confocal; p<0.0001).

Images of *ex vivo* mouse columnar colon epithelium are shown in Fig. 6. The confocal image revealed significant background rejection, which was most prominent in the lumens. In addition, individual nuclei on the glandular walls were more readily resolved. To evaluate this difference, intensity profiles were plotted for two line scans as shown in Figs. 6(c) and 6(d) (corresponding to white lines on the left and right, respectively). Regions for the glandular walls and lumens were identified and indicated by the brackets; the contrast was quantified by comparing the average intensity of the glandular wall (maxima) to the lumen (minima). The ratio between the two was calculated for 12 pairs of maxima and minima in two line scans. The resulting gland-to-lumen ratio was approximately twice as high in the confocal as in the standard HRME (1.57±0.25 in standard and 3.20±0.84 in confocal; p<0.0001).

## 4 Discussion and Conclusion

In this study, we report the development and *ex vivo* validation of a low-cost confocal microendoscope that integrates a digital light modulator and a CMOS sensor in a compact design. The optical sectioning capability of the confocal HRME improved the visualization of cell architecture, especially in crowded regions. In addition, quantitative analysis reveals an enhancement in parameters, such as the nuclear-to-cytoplasmic ratio, which can potentially facilitate automated objective diagnosis based on cell density and morphology.^20^^,^^21^

The current setup reduces the complexity and cost of confocal imaging by eliminating the need for mechanical scanning. A tradeoff is a reduced frame rate limited by the CMOS and DLP scanning speed. In trigger mode, the CMOS sensor used in this research works at a frame rate of 7 to 8 fps; since two consecutive frames need to be averaged, the frame rate is further cut down to 3 to 4 fps. While this is not problematic for imaging *ex vivo* specimens as seen here, motion artifacts will likely impair image quality *in vivo*. For these applications, the imaging speed can be accelerated with a faster CMOS sensor and DLP. The frame rate can also be potentially doubled if the illumination artifacts on a single frame can be erased by adjusting the local gain based on the exposure distribution.

While *ex vivo* imaging confirmed the benefits of optical sectioning, the axial resolution of the confocal HRME can be further improved to better reject background signal. Measured axial profiles suggest that the system sectioning performance could be increased by reducing the detection slit size, while it is ultimately limited by the core spacing of the fiber bundle. This can be realized in the future with a faster DLP supporting sequential projection of added patterns, although it may increase the system cost. Alternatively, if there is only minimal sample motion, the out-of-focus signal can be further reduced at the expense of a lower frame rate. For example, the residual background signal during line-scanning can be recorded with a detection slit offset, and an image subtraction offers an axial resolution comparable to a point-scanning confocal microscope.^18^^,^^27^ In a different approach, multifocal scanning can be introduced via the DLP and consecutive images can be multiplexed to reconstruct a confocal image with improved background rejection.^28^^,^^29^

In conclusion, the work here presents an affordable and portable microendoscope capable of confocal imaging. The versatility of a digital illumination modulator and an electrical detection aperture permits convenient implementation and evaluation of varied optical arrangements. Further optimization is required to increase the current frame rate for *in vivo* applications. Future work will also be needed to explore the potential benefits of optical sectioning in a variety of pathological conditions.

## References

[1] Goetz M. Watson A. Kiesslich R., “Confocal laser endomicroscopy in gastrointestinal diseases,” J. Biophotonics 4, 498–508 (2011). 10.1002/jbio.v4.7/8 21567975

[r2] Muldoon T. J. et al., “High-resolution imaging in Barrett’s esophagus: a novel, low-cost endoscopic microscope,” Gastrointest. Endoscopy 68, 737–744 (2008). 10.1016/j.gie.2008.05.018 PMC2869299 18926182

[r3] Canto M. I. et al., “In vivo endomicroscopy improves detection of Barrett’s esophagus–related neoplasia: a multicenter international randomized controlled trial (with video),” Gastrointest. Endoscopy 79, 211–221 (2014). 10.1016/j.gie.2013.09.020 PMC4668117 24219822

[r4] Chang S. S. et al., “High resolution microendoscopy for classification of colorectal polyps,” Endoscopy 45, 553–559 (2013). ENDCAM 10.1055/s-00000012 23780842

[r5] Nguyen D. L. et al., “The current and future role of endomicroscopy in the management of inflammatory bowel disease,” Ann. Gastroenterol. 28, 331–336 (2015). 26130373 PMC4480169

[6] Park J. C. et al., “Probe-based confocal laser endomicroscopy in the margin delineation of early gastric cancer for endoscopic submucosal dissection,” J. Gastroenterol. Hepatol. 32(5), 1046–1054 (2016). 10.1111/jgh.13635 27862291

[7] Quinn M. K. et al., “High-resolution microendoscopy for the detection of cervical neoplasia in low-resource settings,” PLoS One 7, e44924 (2012). POLNCL 1932-6203 10.1371/journal.pone.0044924 23028683 PMC3445555

[8] Napoléon B. et al., “A novel approach to the diagnosis of pancreatic serous cystadenoma: needle-based confocal laser endomicroscopy,” Endoscopy 47, E26–E27 (2015). ENDCAM 10.1055/s-00000012 25325684

[9] Wellikoff A. S. et al., “Comparison of in vivo probe-based confocal laser endomicroscopy with histopathology in lung cancer: a move toward optical biopsy,” Respirology 20, 967–974 (2015). 10.1111/resp.2015.20.issue-6 26094505

[10] Neumann H. et al., “Confocal laser endomicroscopy: technical advances and clinical applications,” Gastroenterology 139, 388–392 (2010). GASTAB 0016-5085 10.1053/j.gastro.2010.06.029 20561523

[11] Muldoon T. J. et al., “High-resolution imaging in Barrett’s esophagus: a novel, low-cost endoscopic microscope,” Gastrointest. Endoscopy 68, 737–744 (2008). 10.1016/j.gie.2008.05.018 PMC2869299 18926182

[12] Bozinovic N. et al., “Fluorescence endomicroscopy with structured illumination,” Opt. Express 16, 8016–8025 (2008). OPEXFF 1094-4087 10.1364/OE.16.008016 18545511

[13] Sabharwal Y. S. et al., “Slit-scanning confocal microendoscope for high-resolution in vivo imaging,” Appl. Opt. 38, 7133–7144 (1999). APOPAI 0003-6935 10.1364/AO.38.007133 18324260

[14] Kyrish M. et al., “Needle-based fluorescence endomicroscopy via structured illumination with a plastic, achromatic objective,” J. Biomed. Opt. 18, 096003 (2013). JBOPFO 1083-3668 10.1117/1.JBO.18.9.096003 24002190 PMC3759804

[r15] Keahey P. et al., “Differential structured illumination microendoscopy for in vivo imaging of molecular contrast agents,” Proc. Natl. Acad. Sci. U. S. A. 113, 10769–10773 (2016). 10.1073/pnas.1613497113 27621464 PMC5047189

[r16] Keahey P. A. et al., “Optimizing modulation frequency for structured illumination in a fiber-optic microendoscope to image nuclear morphometry in columnar epithelium,” Biomed. Opt. Express 6, 870 (2015). BOEICL 2156-7085 10.1364/BOE.6.000870 25798311 PMC4361441

[r17] Koucky M. H. Pierce M. C., “Axial response of high-resolution microendoscopy in scattering media,” Biomed. Opt. Express 4, 2247–2256 (2013). BOEICL 2156-7085 10.1364/BOE.4.002247 24156080 PMC3799682

[r18] Hughes M. Yang G., “Line-scanning fiber bundle endomicroscopy with a virtual detector slit,” Biomed. Opt. Express 7, 2257 (2016). BOEICL 2156-7085 10.1364/BOE.7.002257 27375942 PMC4918580

[19] Schlosser C. et al., “Fluorescence confocal endomicroscopy of the cervix: pilot study on the potential and limitations for clinical implementation,” J. Biomed. Opt. 21, 126011 (2016). JBOPFO 1083-3668 10.1117/1.JBO.21.12.126011 27999860 PMC8357321

[20] Shin D. et al., “Quantitative analysis of high-resolution microendoscopic images for diagnosis of esophageal squamous cell carcinoma,” Clin. Gastroenterol. Hepatol. 13, 272–279 (2015). 10.1016/j.cgh.2014.07.030 25066838 PMC4305504

[21] Quang T. et al., “A tablet-interfaced high-resolution microendoscope with automated image interpretation for real-time evaluation of esophageal squamous cell neoplasia,” Gastrointest. Endoscopy 84, 834–841 (2016). 10.1016/j.gie.2016.03.1472 PMC5045314 27036635

[22] Rouse A. R. et al., “Design and demonstration of a miniature catheter for a confocal microendoscope,” Appl. Opt. 43, 5763–5771 (2004). APOPAI 0003-6935 10.1364/AO.43.005763 15540433

[23] ASGE Technology Committee, “Confocal laser endomicroscopy,” Gastrointest. Endosc. 80, 928–938 (2014). 10.1016/j.gie.2014.06.021 25442092

[24] Mei E. et al., “A line scanning confocal fluorescent microscope using a CMOS rolling shutter as an adjustable aperture,” J. Microsc. 247, 269–276 (2012). JMICAR 0022-2720 10.1111/j.1365-2818.2012.03642.x 22906014

[25] Muller M. S., “A pico projector source for confocal fluorescence and ophthalmic imaging,” Proc. SPIE 8254, 825408 (2012). 10.1117/12.909574 PMC3824265 24236223

[26] Muller M. S. et al., “Non-mydriatic confocal retinal imaging using a digital light projector,” Proc. SPIE 9376, 93760E (2015). 10.1117/12.2077704 PMC4749182 26877576

[27] Poher V. et al., “Improved sectioning in a slit scanning confocal microscope,” Opt. Lett. 33, 1813–1815 (2008). OPLEDP 0146-9592 10.1364/OL.33.001813 18709096

[28] Bewersdorf J. Pick R. Hell S. W., “Multifocal multiphoton microscopy,” Opt. Lett. 23, 655–657 (1998). OPLEDP 0146-9592 10.1364/OL.23.000655 18087301

[29] York A. G. et al., “Resolution doubling in live, multicellular organisms via multifocal structured illumination microscopy,” Nat. Methods 9, 749–754 (2012).1548-7091 10.1038/nmeth.2025 22581372 PMC3462167

